# Identification of Novel Biallelic *TLE6* Variants in Female Infertility With Preimplantation Embryonic Lethality

**DOI:** 10.3389/fgene.2021.666136

**Published:** 2021-06-11

**Authors:** Manyu Zhang, Chunyu Liu, Beili Chen, Mingrong Lv, Huijuan Zou, Yajing Liu, Yang Gao, Tianjuan Wang, Qiong Xing, Yutong Zhu, Huan Wu, Zhiguo Zhang, Ping Zhou, Zhaolian Wei, Xiaojin He, Yuping Xu, Yunxia Cao

**Affiliations:** ^1^Reproductive Medicine Center, Department of Obstetrics and Gynecology, The First Affiliated Hospital of Anhui Medical University, Hefei, China; ^2^NHC Key Laboratory of Study on Abnormal Gametes and Reproductive Tract Anhui Medical University, Hefei, China; ^3^Key Laboratory of Population Health Across Life Cycle, Anhui Medical University, Ministry of Education of the People’s Republic of China, Hefei, China; ^4^Obstetrics and Gynecology Hospital, NHC Key Laboratory of Reproduction Regulation (Shanghai Institute of Planned Parenthood Research), State Key Laboratory of Genetic Engineering at School of Life Sciences, Fudan University, Shanghai, China; ^5^Shanghai Key Laboratory of Female Reproductive Endocrine Related Diseases, Shanghai, China; ^6^State Key Laboratory of Reproductive Medicine, Center for Global Health, School of Public Health, Nanjing Medical University, Nanjing, China; ^7^Anhui Province Key Laboratory of Reproductive Health and Genetics, Hefei, China; ^8^Biopreservation and Artificial Organs, Anhui Provincial Engineering Research Center, Anhui Medical University, Hefei, China

**Keywords:** preimplantation embryonic lethality, transducin-like enhancer of split 6 (TLE6), variant, whole-exome sequencing, oocyte donation

## Abstract

Preimplantation embryonic lethality is a rare cause of primary female infertility. It has been reported that variants in the transducin-like enhancer of split 6 (*TLE6*) gene can lead to preimplantation embryonic lethality. However, the incidence of *TLE6* variants in patients with preimplantation embryonic lethality is not fully understood. In this study, we identified four patients carrying novel biallelic *TLE6* variants in a cohort of 28 patients with preimplantation embryonic lethality by whole-exome sequencing and bioinformatics analysis, accounting for 14.29% (4/28) of the cohort. Immunofluorescence showed that the TLE6 levels in oocytes from patients were much lower than in normal control oocytes, suggesting that the variants result in the lower expression of the TLE6 protein in oocytes. In addition, a retrospective analysis showed that the four patients underwent a total of nine failures of *in vitro* fertilization and intracytoplasmic sperm injection attempts, and one of them became pregnant on the first attempt using donated oocytes. Our study extends the genetic spectrum of female infertility caused by variants in *TLE6* and further confirms previously reported findings that TLE6 plays an essential role in early embryonic development. In such case, oocyte donation may be the preferred treatment.

## Introduction

Infertility affects about 10–15% of couples worldwide and has become an increasingly common health problem (Tamrakar and Bastakoti, 2019). In recent years, assisted reproductive technology (ART) has become an important treatment for many women suffering from infertility. Recent evidence from ART and embryo research suggests that preimplantation embryonic lethality (PEL) (OMIM:616814) may be a rare cause of primary female infertility (Yatsenko and Rajkovic, 2019). Women with PEL have normal ovarian follicle development and ovulation while suffering from recurrent failures of *in vitro* fertilization (IVF) and intracytoplasmic sperm injection (ICSI) attempts due to fertilization failure and early embryonic arrest. It is challenging to identify genes that cause preimplantation embryonic lethality in humans.

Maternal effect genes (MEGs) are vital during embryonic cleavage stages. After fertilization, the zygotic genome is transcriptionally quiescent, and early embryo development relies on MEGs that encode many of the RNAs and proteins required for early divisions, chromatin remodeling, epigenetic reprogramming, and transcriptional activation cascades (Jukam et al., 2017). Subcortical maternal complex (SCMC), present in the oocytes and early embryos, contains multiple proteins encoded by MEGs and has been identified as being important for preimplantation mouse embryogenesis (Lu et al., 2017). The SCMC appears to be functionally conserved throughout mammalian species. It consists of at least eight proteins, including OOEP, NLRP5, TLE6, KHDC3L, PADI6, ZBED3, NLRP2, and NLRP7 (Zhu et al., 2015; Xu et al., 2016; Mahadevan et al., 2017; Monk et al., 2017; Gao et al., 2018). In mice, knockout of certain MEGs, namely, *Nlrp2*, *Mater*, *Padi6*, *Floped*, and *Tle6*, leads to infertility or subfertility owing to embryonic arrest (Tong et al., 2000; Li et al., 2008; Yurttas et al., 2008; Yu et al., 2014; Mahadevan et al., 2017). Recently, some MEGs have been identified in humans by way of whole-exome sequencing (WES) in a limited number of clinical cases. For example, *TLE6* (OMIM: 612399) variants have been shown to result in the earliest known PEL in human (Alazami et al., 2015). Furthermore, biallelic variants in *PATL2* (OMIM: 614661), *WEE2* (OMIM: 614084), *PADI6* (OMIM: 610363), *NLRP5* (OMIM: 609658), and *NLRP2* (OMIM: 609364) have been identified as the causes of a spectrum of PEL phenotypes, including oocyte maturation arrest, fertilization failure, and early embryonic arrest (Xu et al., 2016; Chen et al., 2017; Sang et al., 2018; Mu et al., 2019). However, variants in these genes can only explain a few cases, and the genetic basis of PEL is still largely unclear.

In the current study, we identified novel biallelic variants in *TLE6* in four patients (14.29%, 4/28) from three unrelated families in a small cohort of 28 women affected with PEL by WES, and these variants were confirmed by Sanger sequencing. In addition, immunofluorescence showed that the variants significantly reduced the amount of TLE6 protein in the oocytes from patients. These findings expand the variant spectrum of *TLE6*.

## Materials and Methods

### Study Subjects

We recruited 28 women affected with PEL from the First Affiliated Hospital of Anhui Medical University, between January 2018 and November 2020. All of the 28 infertile women recruited in our study satisfied the following enrolled criteria for PEL: (1) women aged 20–40 years were diagnosed with primary infertility; (2) normal ovulatory status and the morphology of the oocytes without obvious abnormalities; (3) more than once failure of IVF/ICSI cycles caused by embryonic arrest (no high-quality blastocyst). We also excluded women suffering from other causes of infertility, containing chromosomal anomalies, male factors, endometriosis, and endocrinological causes. The clinical characteristics of all 28 infertile women are listed in Supplementary Table 1. Peripheral blood samples for DNA extraction were obtained from the affected individuals, their available family members, and control subjects. This study was approved by the Ethics Committee of the First Affiliated Hospital of Anhui Medical University (number Quick-PJ2020-13-10). All of the subjects gave their informed consent to participate.

### WES, Bioinformatic Analysis, and Sanger Sequencing

Genomic DNA was extracted from peripheral blood of the affected women using DNeasy Blood and Tissue kit (Qiagen, Hilden, Germany). Whole-exome capture was performed using SureSelect^*XT*^ Human All Exon Kit (Agilent Technologies, Santa Clara, CA, United States) following the manufacturer’s instructions, and sequencing was carried out on the HiSeq X-TEN platform (Illumina, San Diego, CA, United States). The Burrows–Wheeler aligner was employed to map the original data to the human genome assembly GRCh37/hg19 (Li and Durbin, 2009). The Picard software was used to delete PCR duplicates and evaluate the quality of variants by obtaining valid reads, valid base, × 90– × 120 coverage ratio, and average coverage depth. We employed the Genome Analysis Toolkit to call and analyze the indels as well as single-nucleotide variants. We filtered out the single-nucleotide variants with read depths less than × 4 (McKenna et al., 2010). Detailed information about the analytical methods was described previously (Lv et al., 2020). Allele frequencies of the variants were searched using the Exome Aggregation Consortium (ExAC) database^1^, the 1000 Genomes Project database^2^, and the Genome Aggregation Database (gnomAD)^3^. The candidate variants and their parental origins were confirmed via Sanger sequencing.

### Evaluation of Embryo Phenotypes

We used a light microscope (IX-71, Olympus, Japan) to observe morphologies of the embryos at different stages of development. Quality of each embryo was evaluated at several predefined timepoints during embryo development according to the conventional guidelines as described previously (Ding et al., 2020).

### Immunofluorescence

Immature (germinal vesicle stage or metaphase I) and unfertilized (metaphase II, MII) oocytes were donated by the affected individuals and control subjects pursuing IVF/ICSI due to male infertility. These immature oocytes were matured *in vitro* following previously described methods (Zou et al., 2020). Oocyte immunofluorescence staining was performed to assess TLE6 localization. Briefly, oocytes from all subjects (control as well as patients) were fixed with 4% paraformaldehyde for 30 min. The oocytes were then processed with a membrane permeabilizing solution [0.1% Triton X-100 in phosphate-buffered saline (PBS)] for 30 min, followed by blocking with 5% donkey serum for 1 h. Furthermore, the oocytes were incubated with a primary mouse anti-TLE6 antibody (1:50, sc-515065, Santa Cruz Biotechnology, Santa Cruz, CA, United States) overnight at 4°C. The oocytes were then incubated with Alexa Fluor 488 AffiniPure Donkey anti-mouse immunoglobulin G (IgG) (H + L) (1:100, 715-545-150, Jackson ImmunoResearch Laboratories, West Grove, PA, United States) and protected from light for 1 h at room temperature. Following this, the oocytes were incubated with 4′,6-diamidino-2-phenylindole (DAPI) for 10 min to label DNA. Finally, the oocytes were visualized using an LSM800 confocal laser-scanning microscope (Zeiss, Jena, Germany).

## Results

### Clinical Characteristics of Patients

**FIGURE 1:**
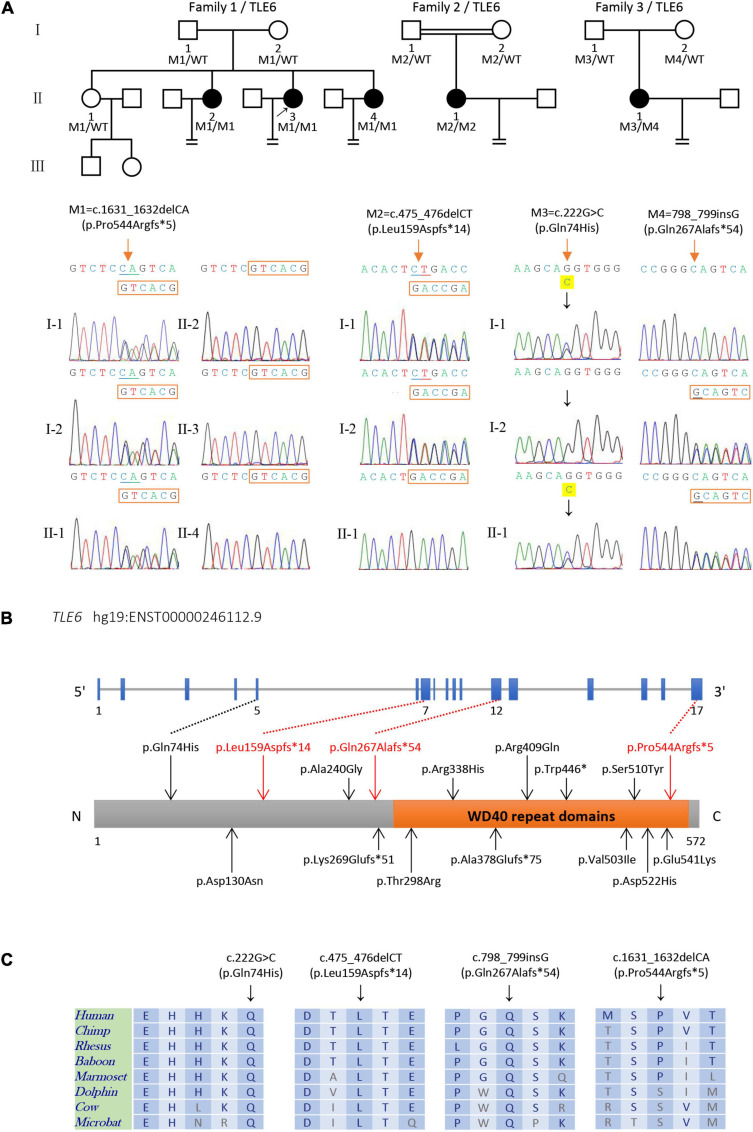
Identification of biallelic *TLE6* variants in the patients and their family members. **(A)** Pedigrees structure of three families harboring variants in *TLE6*, circles represent female patients, squares represent male patients, and black circles denote patients with primary infertility. The double line represents consanguinity, equal signs indicate infertility, and the arrow indicates the proband. Sanger sequencing verification is shown below the pedigrees. Red arrows indicate the mutated positions. **(B)** Schematic representation of the *TLE6* gene and domain structure of its protein product. Black arrows indicate previously reported variants, while red arrows represent the novel variants identified in this study. Orange box represents a cluster of seven WD40 domain repeats. **(C)** Sequence alignment displays conservation of mutant residues in TLE6 among different species.

The clinical information of the four patients carrying the biallelic *TLE6* variants are listed in Table 1, and their family pedigrees are shown in Figure 1A. Their menstrual cycles, karyotypes, transvaginal sonography, and sex hormone levels revealed no abnormalities. Furthermore, their husbands also showed normal semen parameters (sperm concentration, motility, and sperm morphology) as well as karyotypes (Table 1). These patients had been unable to get pregnant despite years of trying.

**TABLE 1:** Clinical laboratory evaluation for the patients carrying variants in *TLE6.*

		**Patients**				
		**II-3, II-4 in family 1**		**II-1 in family 2**	**II-1 in family 3**	
**Gene**		***TLE6***				
cDNA variant^*a*^		c.1631_1632delCA		c.475_476delCT	c.798_799insG sG	c.222G > C
Protein alteration^*b*^		p.Pro544Argfs*5		p.Leu159Aspfs*14	p.Gln267Alafs*54	p.Gln74His
Variant type		Frameshift		Frameshift	Frameshift	Missense
Zygosity		Homozygous		Homozygous	Heterozygous	Heterozygous
Exon		Exon 17		Exon 7	Exon 12	Exon 5
Allele frequency	1KGP	NA		NA	NA	NA
	ExAC	NA		NA	NA	NA
	gnomAD	NA		4.0 × 10^–6^	NA	NA
**Karyotype**						
Female		46, XX	46, XX	46, XX	46, XX	
Male		46, XY	46, XY	46, XY	46, XY	
**Female sex hormone**						
FSH (mIU/ml)		7.34	6.68	8.81	4.19	
LH (mIU/ml)		5.14	3.45	4.64	2.45	
E2 (pmol/L)		138.00	156.63	296.00	169.00	
P (nmol/L)		4.30	0.76	5.62	1.59	
T (nmol/L)		1.40	0.47	2.90	2.80	
PRL (ng/ml)		13.39	17.49	13.32	13.83	
**Male semen parameters**						
Sperm concentration (10^6^/ml)		28.7	59.2	20.5	7.8	
Progressive motility (%) (%)		27.8	47.7	29.8	23.9	
Normal sperm morphology (%)		7	5	4	3	

^a^The GenBank accession numbers of TLE6 is NM_001143986.1. ^b^Full length protein has 572 amino acids. The “*” indicates the termination of protein translation and the number after “*” represents the number of amino acid position corresponding to the stop codon. 1KGP, 1000 Genomes Project; ExAC, Exome Aggregation Consortium; gnomAD, Genome Aggregation Database; NA, not available; FSH, follicle-stimulating hormone; LH, luteinizing hormone; E_2_, estradiol; P, progesterone; T, testosterone; PRL: prolactin.

In family 1, subjects I-1 and I-2 raised four daughters. Three of four sisters (II-2, II-3, and II-4) in this family had infertility for several years. Two of the affected sisters (II-3 and II-4) underwent several IVF/ICSI attempts in the Reproductive Medicine Center of the First Affiliated Hospital of Anhui Medical University. The proband (II-3, 37 years old) had undergone two IVF/ICSI attempts. A total of 44 MII oocytes were retrieved in the two attempts. Only five oocytes were normally fertilized with two-pronucleus (PN) zygotes, while the others were abnormally fertilized with 0PN or degradation on day 1. After cultivation, a majority of her embryos were arrested at the early stages with heavy fragmentation, and only two poor quality blastocysts were available for transfer. Although the proband underwent one frozen–thawed embryo transfer cycle, she failed to obtain a successful pregnancy (Supplementary Table 2).

The other affected sister (II-4, 33 years old) in family 1 had undergone four IVF/ICSI attempts. A total of 55 MII oocytes were retrieved in the four attempts. A majority of oocytes were abnormally fertilized with 0PN; only 10 of them showed normal fertilization with 2PN zygotes on day 1. Most of her embryos were arrested at the early stages accompanied with heavy fragmentation. Only six embryos developed into poor quality blastocysts and were frozen. Patient II-4 underwent three frozen–thawed embryo transfer cycles, none of which was successful (Supplementary Table 2). Finally, she followed an oocyte donation ICSI cycle in which seven donated MII oocytes were obtained. The normal fertilization rate and blastocyst development rate in this cycle were 100.0 and 42.9%, respectively. Patient II-4 obtained three high-quality blastocysts at last and got pregnant on the first embryo transfer.

In family 2, the proband (II-1, 32 years old) had undergone an IVF attempt in which three MII oocytes were obtained. All oocytes had abnormal fertilization with 0PN or degradation on day 1 and were arrested during further blastocyst culture without blastocyst formation (Supplementary Table 2).

In family 3, the proband (II-1, 32 years old) had undergone two IVF/ICSI attempts, which also resulted in 17 retrieved MII oocytes. Most of the oocytes were abnormally fertilized with 0PN or degradation on day 1. Only one of the zygotes formed a poor quality blastocyst, while the others showed developmental arrest on day 3. The patient underwent a frozen–thawed embryo transfer cycle but failed to establish pregnancy (Supplementary Table 2).

### Identification of Biallelic Variants in *TLE6*

We recruited 28 affected individuals with preimplantation embryonic lethality and identified four affected individuals (accounting for 14.29% of the cohort) from three unrelated families carrying biallelic variants *TLE6* (NM_001143986.1) by whole-exome sequencing and bioinformatics analyses. Two (II-3 and II-4 in family 1) of the four affected individuals were sisters from a non-consanguineous family in which three-quarters of the sisters were diagnosed with primary infertility, both of them carrying a homozygous *TLE6* frameshift variant c.1631_1632delCA (p.Pro544Argfs^∗^5). In family 1, Sanger sequencing verified that the parents and one fertile elder sister were heterozygous carriers, while the other three sisters were homozygous for the variant, indicating a recessive inheritance pattern (Figure 1A). Another homozygous frameshift variant in *TLE6* (c.475_476delCT, p.Leu159Aspfs^∗^14) was identified in the proband (II-1 in family 2) from a consanguineous family. Sanger sequencing confirmed that the proband’s parents were both heterozygous carriers (Figure 1A). Moreover, we identified a compound heterozygous variant in *TLE6* (c.222G > C, p.Gln74His; c.798_799insG, p.Gln267Alafs^∗^54) in the proband (II-1 in family 3) from another non-consanguineous family, and the two variants were also inherited from her heterozygous parental carriers, respectively (Figure 1A).

**FIGURE 2:**
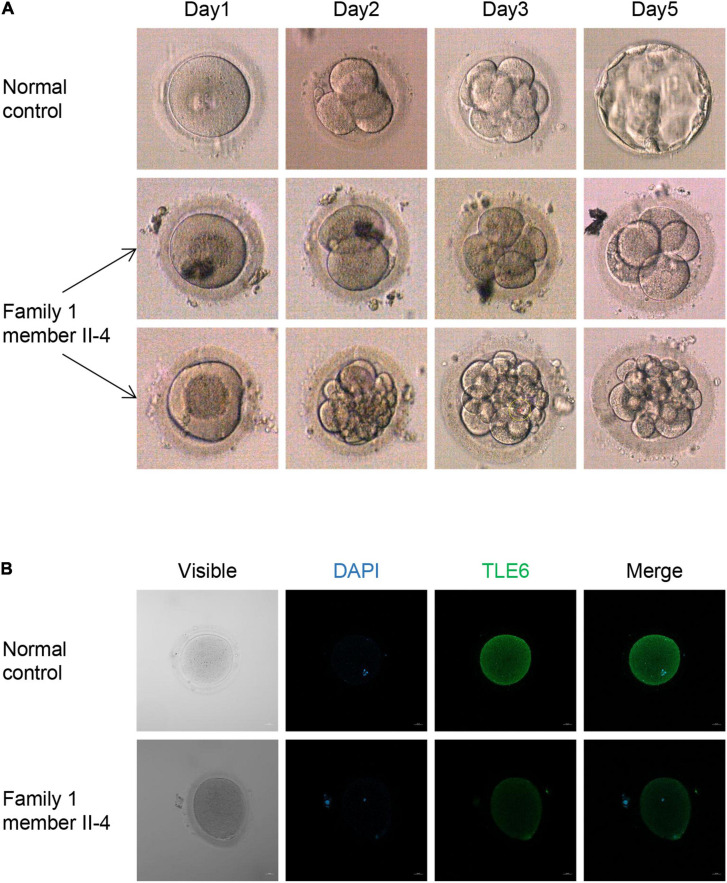
Phenotypes of embryos and oocytes from control and patients. **(A)** Phenotype of control embryos from a subject underwent the ICSI attempt owing to male infertility and embryos from the patient II-4 in family 1. The light microscope was used to observe the morphologies of embryos on days 1, 2, 3, and 5 during cultivation. **(B)** The morphologies and immunofluorescence staining results of an oocyte from the patient II-4 in family 1 and a normal oocyte from a control individual. Oocytes were stained with DAPI (blue) in order to visualize the DNA and immunolabeled with antibodies against TLE6 (green). Scale bar, 20 μm.

These *TLE6* variants were absent in the ExAC database, the 1000 Genomes Project database, and the gnomAD database, except that the *TLE6* frameshift variant c.475_476delCT was found at extremely low allele frequency (4.0 × 10^–6^) in the general population in the gnomAD (Table 1). The three *TLE6* frameshift variants c.1631_1632delCA, c.475_476delCT, and c.798_799insG were all loss-of-function (LoF) variants that caused impaired function of the gene-encoded protein. Positions of the four *TLE6* variants and conservation of mutant residues in their expressed protein among different species are shown in Figure 2. Only the *TLE6* frameshift variant c.1631_1632delCA (p.Pro544Argfs^∗^5) was located within the C-terminal WD40 repeat domain, while the other three variants were to the left of the WD40 repeat domain (Figure 1B). The positions of these four variants are highly conserved in primates (Figure 1C).

### Phenotypic Spectrum of Patients With *TLE6* Variants

We used light microscopy to observe the development and morphology of the embryos from family 1 member II-4 for 5 consecutive days in her last ICSI attempt. Five of the embryos on day 3 were arrested, whereas the others had a high percentage of fragmentation, and all of them failed to form blastocysts (Figure 2A). An immunofluorescence analysis showed that an immunofluorescence signal for TLE6 was observed in the cytoplasm in the control oocytes, similar to previous reports of the localization of other SCMC component proteins in human oocytes, such as PADI6 and NLRP2 (Xu et al., 2016; Mu et al., 2019). However, the TLE6 signal was much weaker in oocytes from the affected individual (II-4 in family 1) compared with control oocytes (Figure 2B). These data suggest that the biallelic variants in *TLE6* result in lower expression of the TLE6 protein in oocytes.

## Discussion

Many infertile patients have experienced several failed attempts of IVF/ICSI. In some of them, ovulatory status is normal and the obtained oocytes look normal, but zygote formation and embryonic development are severely impaired; the phenotypes in these patients including failure of fertilization failure and early embryonic arrest are referred to preimplantation embryonic lethality. In the present study, using WES and bioinformatics analyses, we identified biallelic *TLE6* variants in 4 patients from a cohort of 28 infertile women with PEL, accounting for 14.29% of the cohort.

TLE6, also known as Groucho family member 6 (GRG6), is part of the SCMC that is necessary for mammalian embryonic development (Bajoghli, 2007; Bebbere et al., 2016). Research has shown that high levels of cyclic adenosine monophosphate (cAMP) in the oocyte maintain an increase in cAMP-dependent protein kinase (PKA) activity, which causes meiotic prophase I arrest. Following the luteinizing hormone surge during ovulation, PKA activity reduces due to a decrease in cAMP levels, leading to a resumption of meiosis, while PKA activity increases throughout the process of meiosis from the time of germinal vesicle breakdown (GVBD) until the MII arrest (Duncan et al., 2014). TLE6 is a substrate of PKA during mouse oocyte maturation. Inhibition of PKA activity can lead to delays in GVBD dynamics, abnormal spindle and chromatin structures, and a reduced ability of oocytes to undergo MII (Duncan et al., 2014). Therefore, it has been speculated that the PKA-dependent phosphorylation of TLE6 during GVBD may be relevant to oocyte maturation and subsequent embryonic development. In mouse models, knockout of the *Tle6* gene has no effect on folliculogenesis, oogenesis, and ovulation but instead results in embryonic development arrest at the two-cell stage, and some embryos showed significant fragmentation at embryonic days 2.5 and 3.5, eventually leading to female mice infertility (Li et al., 2008; Yu et al., 2014). Further studies indicate that the SCMC might regulate spindle assembly by controlling formation of the F-actin cytoskeleton to ensure symmetric division of mouse zygotes, while the absence of TLE6 affects the formation of F-actin cytoskeleton due to destruction of the integrity and function of the SCMC and thus results in asymmetric cleavage as well as early embryonic arrest (Yu et al., 2014).

The currently reported mutational and phenotypic spectrum of *TLE6* is listed in Supplementary Table 3. Alazami et al. (2015) first identified a homozygous *TLE6* variant c.1529C > A (p.S510Y) in affected individuals from two Saudi families, which resulted in the earliest known human PEL phenotype, including fertilization failure and early cleavage failure. Their further research showed that the *TLE6* variant not only caused a significant reduction in the PKA-mediated TLE6 phosphorylation but also impaired its binding to other component proteins of the SCMC (Alazami et al., 2015). Wang et al. (2018) found the *TLE6* variant c.1133delC (p.A378Efs^∗^75) being responsible for embryonic developmental arrest on day 3, similar to the phenotype of *Tle6*^*Null*^ mice. Furthermore, another study also found that three patients carrying biallelic *TLE6* variants had fertilization failure and early embryonic arrest in several IVF/ICSI attempts. Two of them obtained a very low number of low-quality embryos but failed to establish pregnancy (Lin et al., 2020). Recently, using time-lapse imaging, Zheng et al. (2021) found that the *TLE6* missense variant c.1564G > C (p.Asp522His) is associated with direct cleavage (zygotes directly cleaved into more than two blastomeres).

In our study, we mainly focused on patients with PEL and did not include cases of oocyte malformation, oocyte maturation arrest, and repeated implantation failure. Therefore, the incidence of *TEL6* variants in our study is higher than other studies (Lin et al., 2020; Maddirevula et al., 2020). Furthermore, phenotypes of infertile women harboring biallelic *TLE6* variants in this study are similar to the previously reported clinical cases (Alazami et al., 2015; Wang et al., 2018; Lin et al., 2020; Zheng et al., 2021). These four patients underwent a total of nine IVF/ICSI attempts in which the number and morphology of retrieved MII oocytes were not clearly abnormal. However, a majority of their oocytes were abnormally fertilized with 0PN or degradation on day 1, and the normal fertilization rate (the 2PN rate) was low (10.3 ± 7.6%). Patients II-4 and II-3 from family 1 had embryonic arrest with a high proportion of embryo fragmentation (36.1 ± 1.1%), while the others only represented embryonic arrest without heavy embryo fragmentation (Supplementary Table 2), indicating that patients harboring different *TLE6* variants showed a broad range of phenotypes, containing poor fertilization, embryonic arrest, and a high rate of embryo fragmentation. Patient II-4 from family 1 got pregnant successfully at the first attempt using donated oocytes. In contrast, subject II-1 in family 1, who had a heterozygous variant in *TLE6*, had normal fertility and two healthy children. Furthermore, in our cohort of control women pursuing IVF/ICSI due to male infertility, we also found that a subject harboring a heterozygous missense variant in TLE6 (c.1067T > C, p.L356P) followed a sperm donation ICSI cycle in which 11 MII oocytes were obtained. The normal fertilization rate and blastocyst development rate in this cycle were 100.0 and 18.2%, respectively. She obtained two high-quality blastocysts and achieved pregnancy on the first embryo transfer. These clinical cases combined with Sanger sequencing can help further understand the inheritance pattern of the *TLE6* mutant gene.

In addition, we used light microscopy for five consecutive days to observe the development and morphology of the embryos from one patient (II-4 in family 1) who carried the homozygous *TLE6* frameshift variant c.1631_1632delCA (p.Pro544Argfs^∗^5) and found that three embryos had a high percentage of fragmentation during culture, whereas the other five embryos were arrested on the third day, showing similar phenotype in embryogenesis between infertile women carrying *TLE6* variants and *Tle6*^*Null*^ female mice. Thus, combined with previous reports, we speculated that the *TLE6* missense/frameshift variants lead to embryo fragmentation by disrupting the F-actin and spindle dynamics. In addition, our study is the first to assess the expression levels of the TLE6 protein in the oocytes of affected individuals. Immunofluorescence staining showed that the biallelic variants in *TLE6* in the present study resulted in the lower expression of TLE6 in the oocytes of affected individuals. From this, we speculate that the lower expression of TLE6 protein might then affect the stability and function of the SCMC, eventually leading to preimplantation embryonic lethality.

There are a few limitations associated with the current study. First, the exact molecular mechanism of PEL could not be completely elucidated owing to the paucity of human oocytes and embryos. It will be worthwhile to study the molecular mechanism using knock-in mice for each variant in the future. Second, sample size of affected women was limited in the present study, so the incidence of *TLE6* variants in patients with PEL requires further research.

In conclusion, this study extends the spectrum of variants in *TLE6* and suggests that biallelic *TLE6* variants are likely responsible for preimplantation embryonic lethality. Oocyte donation may be the best ART available right now for patients harboring biallelic *TLE6* variants. Interestingly, very recently, nuclear transfer has been proposed to overcome embryo developmental arrest, as well as injection of wild-type *WEE2* cRNA into oocytes has been reported to overcome fertilization failure caused by *WEE2* variants (Sang et al., 2018; Christodoulaki et al., 2021). Further studies need to be performed in the future to explore whether these techniques could also be an option for patients with *TEL6* variants.

## Data Availability Statement

The original contributions presented in the study are included in the article/Supplementary Material, further inquiries can be directed to the corresponding authors.

## Ethics Statement

The studies involving human participants were reviewed and approved by the Ethics Committee of the First Affiliated Hospital of Anhui Medical University. The patients/participants provided their written informed consent to participate in this study. Written informed consent was obtained from the individual(s) for the publication of any potentially identifiable images or data included in this article.

## Author Contributions

MZ wrote the manuscript. CL and ML analyzed the data. BC, HZ, YL, and MZ conducted the experiments. YG, TW, QX, and YZ collected the sample and data. HW, XH, and YX designed and directed the study. ZZ, PZ, and ZW revised the manuscript. YC was responsible for the study supervision. All authors contributed to the article and approved the submitted version.

## Conflict of Interest

The authors declare that the research was conducted in the absence of any commercial or financial relationships that could be construed as a potential conflict of interest.

## Footnotes

1 http://exac.broadinstitute.org/

2 http://www.1000genomes.org/

3 http://gnomad.broadinstitute.org/
