# Supplementary material for: Identification of Novel Biallelic TLE6 Variants in Female Infertility With Preimplantation Embryonic Lethality
Source: Front Genet. 2021 Jun 11;12:666136. doi: 10.3389/fgene.2021.666136 (PMC8226231; doi:10.3389/fgene.2021.666136)
Supplement: Supplementary file 1 [file Data_Sheet_1.docx]

**Supplementary Table S1.** Clinical characteristics of the 28 infertile women recruited in our study

| **Infertile women NO.** | **Age (years)** | **Infertility duration**  **(years)** | **No. of IVF/ICSI cycles** | **No. of oocytes retrieved** | **No. of MII oocytes** | **Fertilization rate (%)** | **Cleavage rate (%)** | **Blastocyst development rate (%)** | **No. of poor quality blastocysts** | **No. of embryos transfer cycles** | **No. of embryos transferred** | **Clinical pregnancy** |
| --- | --- | --- | --- | --- | --- | --- | --- | --- | --- | --- | --- | --- |
| 1 | 29 | 2 | 2 | 12 | 9 | 9/9(100.0) | 8/9(88.9) | 1/9(11.1) | 1 | 1 | 1 | No |
| 2 | 28 | 4 | 2 | 41 | 29 | 7/29(24.1) | 7/7(100.0) | 2/7(28.6) | 2 | 1 | 2 | No |
| 3 | 33 | 12 | 5 | 85 | 61 | 37/61(60.7) | 36/37(97.3) | 7/37(18.9) | 7 | 4 | 7 | No |
| 4 | 30 | 2 | 3 | 22 | 15 | 12/15(80.0) | 12/12(100.0) | 2/12(16.7) | 2 | 1 | 2 | No |
| 5* | 37 | 15 | 2 | 71 | 44 | 17/44(38.6) | 17/17(100.0) | 2/17(11.8) | 2 | 1 | 2 | No |
| 6* | 33 | 9 | 4 | 92 | 55 | 38/55(69.1) | 38/38(100.0) | 6/38(15.8) | 6 | 3 | 6 | No |
| 7 | 33 | 5 | 2 | 36 | 29 | 18/29(62.1) | 11/18(61.1) | 0 | 0 | 0 | 0 | No |
| 8 | 30 | 4 | 2 | 28 | 28 | 24/28(85.7) | 24/24(100.0) | 4/24(16.7) | 4 | 2 | 4 | No |
| 9 | 29 | 7 | 2 | 15 | 13 | 12/13(92.3) | 12/12(100.0) | 2/12(16.7) | 2 | 1 | 2 | No |
| 10 | 31 | 6 | 2 | 41 | 32 | 24/32(75.0) | 22/24(91.7) | 6/24(25.0) | 6 | 3 | 6 | No |
| 11 | 28 | 5 | 3 | 39 | 25 | 21/25(84.0) | 21/21(100.0) | 2/21(9.5) | 2 | 1 | 2 | No |
| 12 | 29 | 5 | 2 | 20 | 17 | 10/17(58.8) | 9/10(90.0) | 1/10(10.0) | 1 | 1 | 1 | No |
| 13 | 31 | 6 | 2 | 63 | 55 | 46/55(83.6) | 43/46(93.5) | 6/46(13.0) | 6 | 3 | 6 | No |
| 14 | 34 | 11 | 2 | 26 | 21 | 18/21(85.7) | 18/18(100.0) | 3/18(16.7) | 3 | 2 | 3 | No |
| 15*^,a^ | 32 | 3 | 1 | 5 | 3 | 1/3(33.3) | 1/1(100.0) | 0 | 0 | 0 | 0 | No |
| 16 | 30 | 5 | 2 | 57 | 39 | 23/39(59.0) | 22/23(95.7) | 4/23(17.4) | 4 | 0 | 0 | No |
| 17 | 34 | 7 | 2 | 20 | 17 | 6/17(35.3) | 3/6(50.0) | 0 | 0 | 0 | 0 | No |
| 18 | 34 | 8 | 2 | 19 | 19 | 16/19(84.2) | 4/16(25.0) | 2/16(12.5) | 2 | 1 | 2 | No |
| 19 | 33 | 3 | 2 | 36 | 22 | 18/22(81.8) | 11/18(61.1) | 0 | 0 | 0 | 0 | No |
| 20 | 34 | 3 | 2 | 16 | 15 | 14/15(93.3) | 14/14(100.0) | 3/14(21.4) | 3 | 1 | 2 | No |
| 21 | 30 | 3 | 2 | 20 | 19 | 19/19(100.0) | 19/19(100.0) | 3/19(15.8) | 3 | 2 | 3 | No |
| 22* | 32 | 2 | 2 | 24 | 17 | 5/17(29.4) | 5/5(100.0) | 1/5(20.0) | 1 | 1 | 1 | No |
| 23 | 31 | 3 | 3 | 27 | 22 | 16/22(72.7) | 16/16(100.0) | 3/16(18.8) | 3 | 2 | 3 | No |
| 24 | 29 | 5 | 3 | 61 | 53 | 44/53(83.0) | 42/44(95.5) | 8/44(18.2) | 8 | 3 | 8 | No |
| 25 | 26 | 2 | 2 | 14 | 10 | 6/10(60.0) | 6/6(100.0) | 0 | 0 | 0 | 0 | No |
| 26 | 32 | 9 | 3 | 22 | 18 | 17/18(94.4) | 9/17(52.9) | 2/17(11.8) | 2 | 1 | 2 | No |
| 27 | 28 | 2 | 2 | 28 | 18 | 16/18(88.9) | 16/16(100.0) | 1/16(6.3) | 1 | 1 | 1 | No |
| 28 | 30 | 2 | 2 | 27 | 22 | 15/22(68.2) | 15/15(100.0) | 2/15(13.3) | 2 | 1 | 1 | No |

^a^ The patient had another failed IVF cycle with embryonic arrest at other hospitals. It is unable to obtain detailed clinical information.

Abbreviation: *, four patients carrying novel biallelic TLE6 variants in the cohort of 28 patients; IVF, in vitro fertilization; ICSI, intracytoplasmic sperm injection; MII, metaphase II.

**Supplementary Table S2.** Oocyte and embryo characteristics of IVF and ICSI attempts for the four patients

| **Patient** | **IVF/**  **ICSI**  **Attempts** | **Retrieved oocytes** | **MII**  **Oocytes** | **Fertilized Outcomes (2PN+1PN+MPN+0PN)** | **Cleaved / Fragmented embryo** | **Embryo Outcomes** | |
| --- | --- | --- | --- | --- | --- | --- | --- |
|  |  |  |  |  |  | **On day 3** | **On day 5 or 6** |
| **II-3 in family 1** | ICSI | 23 | 14 | 1+0+0+13 | 6/0 | 2*4-cell, 4*2-cell | the two 4-cell embryos were further cultured and formed two blastocysts (a grade 3BC blastocyst and a grade 3CB blastocyst) but failed to implant, the others were arrested at the 2-cell stage during in vitro culture |
|  | ICSI | 48 | 30 | 4+0+0+25,  1degeneration | 11/5 | 1*7-cell, 1*6-cell, 2*5-cell, 2*3-cell, 5*2-cell | all embryos were arrested at the 2-cell to 7-cell stage during in vitro culture |
| **II-4 in family 1** | IVF | 21 | 13 | 3+0+0+10 | 11/5 | 2*8-cell, 1*7-cell, 3*6-cell, 1*5-cell, 3*3-cell, 1*2-cell | two 8-cell embryos were further cultured and formed two blastocysts (two grade 3BC blastocysts) but failed to implant, the others were arrested at the 2-cell to 7-cell stage during in vitro culture |
|  | ICSI | 20 | 14 | 3+0+0+11 | 10/2 | 2*6-cell, 3*5-cell, 1*4-cell, 1*3-cell, 3*2-cell | two 6-cell embryos were further cultured and formed two blastocysts (a grade 3BC blastocyst and a grade 3BB blastocyst) but failed to implant, the others were arrested at the 2-cell to 5-cell stage during in vitro culture |
|  | ICSI | 26 | 13 | 2+0+0+11 | 9/4 | 1*8-cell, 3*5-cell, 2*4-cell, 3*3-cell | one 4-cell embryo and one 8-cell embryo were further cultured and formed two blastocysts (two grade 3CB blastocysts) but failed to implant, the others were arrested at the 3-cell to 5-cell stage during in vitro culture |
|  | ICSI | 25 | 15 | 2+0+0+13 | 8/3 | 2*9-cell, 1*8-cell, 1*5-cell, 3*3-cell, 1*2-cell | all embryos were arrested at the 2-cell to 9-cell stage during in vitro culture |
| **II-1 in family 2** | ICSI | 5 | 3 | 0+0+0+2, 1 degeneration | 1/0 | 1*2-cell | the embryo was arrested at the 2-cell stage during in vitro culture |
| **II-1 in family 3** | ICSI | 15 | 8 | 1+0+0+5,  2 degeneration | 1/0 | 1*6-cell | the embryo was arrested at the 6-cell stage during in vitro culture |
|  | ICSI | 9 | 9 | 1+0+0+8 | 4/0 | 2*6-cell, 1*3-cell, 1*2-cell | one 6-cell embryo was further cultured and formed a blastocyst (grade 3BC) but failed to implant, the others were arrested at the 2-cell to 6-cell stage during in vitro culture |

Abbreviation: IVF, in vitro fertilization; ICSI, intracytoplasmic sperm injection; MII, metaphase II; PN, pronucleus; MPN, muti-pronucleus.

**Supplementary Table S3**. Overview of the currently reported the mutational and phenotypic spectrum of *TLE6*

| **Genomic position on chr.19 (bp) (hg19)** | **Transcripts** | **Exon** | **cDNA Change** | **Protein Change** | **Mutation Type** | **Geno**  **type** | **ExAC Allele frequency** | **Phenotypes** | **References** |
| --- | --- | --- | --- | --- | --- | --- | --- | --- | --- |
| 2993572 | NM_001143986.1 | 15 | c.1529C > A | p.Ser510Tyr | missense | hom | NA | fertilization failure | Alazami et al., 2015 |
| 2989671 | NM_001143986.1 | 13 | c.1133delC | p.Ala378Glufs*75 | Frameshift | hom | 4.96E-05 | early embryonic arrest | Wang et al., 2018 |
| 2989765 | NM_001143986.1 | 13 | c.1226G>A | p.Arg409Gln | missense | hom | NA | fertilization failure | Lin et al., 2020 |
| 2994904 | NM_001143986.1 | 17 | c.1621G>A | p.Glu541Lys | missense | hom | NA | early embryonic arrest，failure after low-quality embryos transfer |  |
| 2987083 | NM_001143986.1 | 7 | c.388G>A | p.Asp130Asn | missense | com-het | 8.26E-06 | early embryonic arrest，failure after low-quality embryos transfer |  |
| 2993550 | NM_001143986.1 | 15 | c.1507G>A | p.Val503Ile | missense | com-het | 5.99E-05 | early embryonic arrest，failure after low-quality embryos transfer |  |
| 2989123-2989124 | NM_024760.2 | 11 | c.436_437delAA | p.Lys146Glufs*51 | Frameshift | hom | NA | a high rate of embryo fragmentation | Maddirevula et al., 2020 |
| 2982187 | NM_001143986 | 5 | c.222G>C | p.Gln74His | missense | hom/  com-het | NA | early embryonic arrest，a high rate of embryo fragmentation | Zheng et al., 2021 |
| 2988105 | NM_001143986 | 11 | c.719C>G | p.Ala240Gly | missense | com-het | NA | early embryonic arrest，a high rate of embryo fragmentation |  |
| 2994043 | NM_001143986 | 16 | c.1564G>C | p.Asp522His | missense | hom | NA | early embryonic arrest，direct cleavage，  a high rate of embryo fragmentation |  |
| 2989552 | NM_001143986 | 13 | c.1013G>A | p.Arg338His | missense | com-het | 3.43E-05 | early embryonic arrest，a high rate of embryo fragmentation |  |
| 2991933 | NM_001143986 | 14 | c.1337G>A | p.Trp446* | nonsense | com-het | NA | early embryonic arrest，a high rate of embryo fragmentation |  |
| 2989211 | NM_001143986 | 12 | c. 893C>G | p.Thr298Arg | missense | hom | NA | early embryonic arrest，a high rate of embryo fragmentation |  |
